# Almost automorphic solutions for shunting inhibitory cellular neural networks with time-varying delays

**DOI:** 10.1186/s40064-015-1507-4

**Published:** 2015-11-24

**Authors:** Changjin Xu, Maoxin Liao

**Affiliations:** 1 Guizhou Key Laboratory of Economics System Simulation, Guizhou University of Finance and Economics, Guiyang, 550004 People’s Republic of China; 2 School of Mathematics and Physics, University of South China, Hengyang, 421001 People’s Republic of China

**Keywords:** Shunting inhibitory cellular neural networks, Almost automorphic solution, Exponential stability, Time-varying delay, 39A60, 39A12

## Abstract

This paper is concerned with the shunting inhibitory cellular neural networks with time-varying delays. Under some suitable conditions, we establish some criteria on the existence and global exponential stability of the almost automorphic solutions of the networks. Numerical simulations are given to support the theoretical findings.

## Background

It is well known that shunting inhibitory cellular neural networks with delay have been successfully applied in variety of areas such as signal processing, pattern recognition, chemical processes, nuclear reactors, biological systems, static image processing, associative memories, optimization problems and so on (Roska and Chua 1992; Chua and Yang 1988a, b; Chua and Roska 1990; Zhang and Shao 2013). In the past decades, there have been extensive results on the dynamical behavior of shunting inhibitory cellular neural networks networks such as the existence and stability of equilibrium points, periodic solutions, almost periodic solutions and anti-periodic solutions, etc. We refer the reader to (Wang et al. 2014a, b; Song et al. 2012; Fan and Shao 2010; Li and Wang 2012; Xia et al. 2007; Peng and Wang 2013; Bouzerdoum and Pinter 1993; Chen and Zhao 2008; Xia et al. 2007; Shao 2008; Yang and Cao 2007; Zhang 2013; Huang et al. 2010).

In particular, we shall point out that almost periodicity is universal than periodicity in real word, moreover, almost automorphic functions, which were introduced by Bochner, are much more general than almost periodic functions. The almost automorphic solutions have potential applications in various fields such as linear and nonlinear evolution equations, integro-differential and functional-differential equations, dynamical systems and so on (Cuevas et al. 2012; N’Gérékata 2005). Almost automorphic solutions in the context of differential equations were studied by several authors. We refer the reader to (Hilger 1990; N’Guérékata 2004, 2005; Goldstein and N’Guérékata 2005; Ezzinbi et al. 2007; Chérif and Nahia 2013; Chérif 2014; Wang and Li 2013; Lizama and Mesquita 2013). However, to the best of our knowledge, there are very few papers published on the almost automorphic solutions of shunting inhibitory cellular neural networks with time-varying delays (Li and Yang 2014; Abbas et al. 2014).

Inspired by the discuss above, in this paper, we consider the following shunting inhibitory cellular neural networks with time-varying delays

1 $$\begin{aligned} {x}_{ij}^{'}(t)= & {} -a_{ij}(t)x_{ij}(t)+\sum _{C_{kl}\in N_r(i,j)} C_{ij}^{kl}(t)f(x_{kl}(t-\tau _{kl}(t)))x_{ij}(t)\nonumber \\&+\sum _{C_{kl}\in N_q(i,j)} B_{ij}^{kl}(t)\int _0^\infty K_{ij}(u)g(x_{kl}(t-u))du x_{ij}(t)+L_{ij}(t), \end{aligned}$$

where $C_{ij}$ denotes the cell at the (*i*, *j*) position of the lattice. The *r*-neighborhood $N_r(i,j)$ of $C_{ij}$ is given as

2 $$\begin{aligned} N_r(i,j)=\{C_{kl}:\max (|k-i|,|l-j|)\le r, \quad 1\le k\le m, 1\le l\le n\}, \end{aligned}$$

where $i=1,2,\ldots ,m,j=1,2,\ldots ,n,$ $N_q(i,j)$ is similarly specified, $x_{ij}$ is the activity of the cell $C_{ij}$, $L_{ij}(t)$ is the external input to $C_{ij}$, the function $a_{ij}(t)>0$ represents the passive decay rate of the cell activity, $C_{ij}^{kl}$ and $B_{ij}^{kl}$ are the connection or coupling strength of postsynaptic activity of the cell transmitted to the cell $C_{ij}$, and the activity functions *f*(.) and *g*(.) are continuous functions representing the output or firing rate of the cell $C_{kl}$, and $\tau _{kl}(t)\ge 0$ corresponds to the transmission delay, the kernel $K_{ij}$ is a piecewise continuous integrable function and satisfies

$$\begin{aligned} \int _{-\infty }^tK_{ij}(t-s)ds=1, \int _0^\infty K_{ij}(s)e^{\alpha s}ds<+\infty ,\quad \alpha >0. \end{aligned}$$

It is easy to see that system (1) is equivalent to the form

3 $$\begin{aligned} {x}_{ij}^{'}(t)= & {} -a_{ij}(t)x_{ij}(t)+\sum _{C_{kl}\in N_r(i,j)} C_{ij}^{kl}(t)f(x_{kl}(t-\tau _{kl}(t)))x_{ij}(t)\nonumber \\&+\sum _{C_{kl}\in N_q(i,j)} B_{ij}^{kl}(t)\int _{-\infty }^t K_{ij}(t-u)g(x_{kl}(u))du x_{ij}(t)+L_{ij}(t). \end{aligned}$$

The main aim of this paper is to establish a set of sufficient conditions for the existence and exponential stability of almost automorphic solutions for model (3).

The remainder of the paper is organized as follows. In "Preliminary results", we introduce the basic properties of almost automorphic functions, some necessary notations, definitions and preliminaries which will be used later. In "Existence of almost automorphic solutions" , we present some sufficient conditions for the existence of almost automorphic solutions of (3). Some sufficient conditions on the global exponential stability of almost automorphic solutions of (3) are established in "Exponential stability of almost automorphic solutions". An example is given to illustrate the effectiveness of the obtained results in "Numerical example" . A brief conclusion is drawn in "Conclusions".

## Preliminary results

In this section, we would like to recall some basic definitions and lemmas related to the concept of almost automorphy which shall come into play later on.

### **Definition 2.1**

(*Bochne*r 1962) A continuous function $f : \mathbb {R}\rightarrow \mathbb {R}^n$ is said to be almost automorphic if for every sequence of real numbers $(s_n^{'})_{n\in \mathbb {N}}$, there exists a subsequence $(s_n)_{n\in \mathbb {N}}$ such that $g(t) :=\lim _{n\rightarrow \infty }f (t+s_n)$ is well defined for each $t \in \mathbb {R}$, and $\lim _{n\rightarrow \infty }g (t-s_n)=f(t)$ for each $t\in \mathbb {R}.$

### *Remark 2.1*

(Chérif 2014) Note that the function *g* in definition above is measurable but not necessarily continuous. Moreover, if *g* is continuous, then f is uniformly continuous. Besides, if the convergence above is uniform in $t\in \mathbb {R},$ then *f* is almost periodic. Denote by $AA(\mathbb {R},\mathbb {R}^n)$ the collection of all almost automorphic functions, then

$$\begin{aligned} AP(\mathbb {R},\mathbb {R}^n)\subset AA(\mathbb {R},\mathbb {R}^n) \subset BC(\mathbb {R},\mathbb {R}^n), \end{aligned}$$

where $AP(\mathbb {R},\mathbb {R}^n)$ and $BC(\mathbb {R},\mathbb {R}^n)$ are respectively the collection of all almost periodic functions and the set of bounded continuous functions from $\mathbb {R}$ to $\mathbb {R}^n.$

### **Lemma 2.1**

(N’Guérékata 2005)* For all* $f,f_1,f_2\in AA(\mathbb {R},\mathbb {R}^n)$ *, one has*

1. $$f_1+f_2\in AA(\mathbb {R},\mathbb {R}^n)$$.
2. $\lambda f \in AA(\mathbb {R},\mathbb {R}^n)$ *for any scalar* $\lambda \in \mathbb {R}$.
3. $f_\alpha \in AA(\mathbb {R},\mathbb {R}^n)$,* where* $f_\alpha : \mathbb {R}\rightarrow X$ *is defined by* $f_\alpha (.)=f(.+\alpha ).$
4. *Let* $f\in AA(\mathbb {R},\mathbb {R}^n)$ *, then the range* $R_f:=\{f(t),t\in \mathbb {R}\}$ *is relatively compact in X, thus* *f is bounded in norm.*
5. *If* $f_n\rightarrow f$ *uniformly on* $\mathbb {R}$ *, where* $f_n\in AA(\mathbb {R},\mathbb {R}^n)$ *, then* $f\in AA(\mathbb {R},\mathbb {R}^n)$.
6. $(AA(\mathbb {R},\mathbb {R}^n), ||.||_\infty )$ *is a Banach space.*

### **Definition 2.2**

A function $f\in C(\mathbb {R} \times \mathbb {R}^n, \mathbb {R}^n)$ is said to be almost automorphic in $t\in \mathbb {R}$ for each $x\in X$ if for every sequence of real numbers $(s_n^{'})_{n\in \mathbb {N}}$, there exists a subsequence $(s_n)_{n\in \mathbb {N}}$ such that $g(t, x):= \lim _{n\rightarrow \infty } f(t + s_n, x)$ is well defined for each $t\in \mathbb {R}$, $x\in \mathbb {R}^n$ and $\lim _{n\rightarrow \infty } g(t-s_n, x) = f (t, x)$ for each $t\in \mathbb {R}$, $x\in \mathbb {R}^n$. The collection of such functions will be denoted by $AA(\mathbb {R}\times \mathbb {R}^n,\mathbb {R}^n).$

### **Lemma 2.2**

(Diagana et al. 2008)* Let* $f: \mathbb {R}\times \mathbb {R}^n\rightarrow \mathbb {R}^n$ *be an almost automorphic function in* $t\in \mathbb {R}$ *for each* $x\in \mathbb {R}^n$ *and assume that f satisfies a Lipschitz condition in* *x uniformly in* $t\in \mathbb {R}$.* Let* $\varphi : \mathbb {R}\rightarrow \mathbb {R}^n$ *be an almost automorphic function. Then the function* $\phi : t\mapsto \phi (t)= f (t, \varphi (t))$ *is almost automorphic.*

### **Definition 2.3**

The almost automorphic solution $x_{ij}(.)=(x_{11}(.), x_{12}(.),\ldots , x_{mn}(.))$ of SICNNs is said to be globally exponentially stable, if, for any solution $x(.)=(x_{11}(.), x_{12}(.),\cdots ,x_{mn}(.))$, there exist constants $M>0$ and $\mu >0$ such that for all $t\in \mathbb {R}$,

$$\begin{aligned} ||x^*(t)-x(t)||\le Me^{-\mu t}. \end{aligned}$$

### **Lemma 2.3**

(Hale 1977) (The upper-right Dini derivative) *Let* $f: \mathbb {R} \rightarrow \mathbb {R}$ *be a continuous function, then the upper-right Dini derivative* $\frac{D^{+}f(t)}{dt}$ *is defined by*

$$\begin{aligned} \frac{D^{+}f(t)}{dt}= \overline{\lim _{h\rightarrow 0^{+}}} \frac{f(t + h)-f(t)}{h}. \end{aligned}$$

### *Remark 2.2*

(Abbas et al. 2014) The upper-right Dini derivative $\frac{D^{+}f(t)}{dt}$ of |*f*(*t*)| is given by

$$\begin{aligned} \frac{D^{+}V|f(t)|}{dt}= sign(f(t))\frac{df(t)}{dt}, \end{aligned}$$

where sign(.) is the signum function.

## Existence of almost automorphic solutions

In this section, we will establish sufficient conditions on the existence of almost automorphic solutions of (1). Denote

$$\begin{aligned} \Lambda =\{11,12,1n,21,22,\ldots ,2n,mn\}, \quad \tau =\max _{1\le k\le m,1\le l\le n}\{\tau _{kl}(t)\}. \end{aligned}$$

Throughout this paper, we make the assumptions as follows.

1. There exists constants $L_f>0,L_g>0,\,M_f>0$ and $M_g>0$ such that for all $u, v\in \mathbb {R}$,
  $$\begin{aligned} |f(u)-f(v)|\le L_f|u-v|,|g(u)-g(v)|\le L_g|u-v|, \, |f(u)|\le M_f, \quad |g(u)|\le M_g. \end{aligned}$$
  Furthermore, $$f(0)=g(0)=0.$$
2. For $ij\in \Lambda ,$ $L(.)=(L_{11}(.),L_{12}(.),\cdots ,L_{mn}(.))\in AA(\mathbb {R}, \mathbb {R}^{m+n})$ and $a_{ij}(t), C_{ij}^{kl}$ and $B_{ij}^{kl}$ all almost automorphic.
3. For $$ij\in \Lambda ,$$
  $$\begin{aligned} \gamma =\max _{ij\in \Lambda }\sup _{t\in \mathbb {R}}\Bigg \{\frac{\sum _{C_{kl}\in N_r(i,j)} |C_{ij}^{kl}(t)|L_f+\frac{M}{u}\sum _{C_{kl}\in N_q(i,j)}|B_{ij}^{kl}(t)|L_g}{a^{-}}\Bigg \}<1, \quad \frac{||L||_\infty }{a^{-}(1-\gamma )}<1, \end{aligned}$$
  where $$a_{ij}^{-}=\min _{t\in \mathbb {R}}a_{ij}(t), a^{-}=\min _{ij\in \Lambda }a_{ij}^{-}.$$
4. For $$ij\in \Lambda ,$$ $$\max _{ij\in \Lambda }\sup _{s\in \mathbb {R}}\Big \{\frac{\Pi _{ij}}{a^{-}}\Big \}<1,$$ where
  $$\begin{aligned} \Pi _{ij}=\sum _{C_{kl}\in N_r(i,j)} |C_{ij}^{kl}(s)|(M_f+L_f)+\sum _{C_{kl}\in N_q(i,j)} |B_{ij}^{kl}(s)|\Bigg (1+\frac{||L||_\infty }{a^{-}(1-\gamma )}\Bigg )L_g \int _0^\infty |K_{ij}(u)|du. \end{aligned}$$
5. The kernel $K_{ij}(.)$ is almost automorphic and there exist $M>0$ and $u>0$ such that
  $$\begin{aligned} |K_{ij}(t)|\le Me^{-ut}. \end{aligned}$$

### **Lemma 3.1**

*Suppose that assumptions (H1) and (H5) hold and* $x_{ij}(.)\in AA(\mathbb {R},\mathbb {R})$ *, then*

$$\begin{aligned} \phi :t\mapsto \int _{-\infty }^t K_{ij}(t-s)g(x_{kl}(s))ds \end{aligned}$$

*belongs to* $$AA(\mathbb {R},\mathbb {R})$$.

### *Proof*

By the composition theorem of almost automorphic functions (N’Guérékata 2005), the functions $\psi : s\mapsto g(x_{kl}(s))$ belongs to $AA(\mathbb {R},\mathbb {R})$ whenever $x_{kl}\in AA(\mathbb {R},\mathbb {R}^{m+n})$. Now, let $(s_n^{'})$ be a sequence of real numbers. By (H5), we can extract a subsequence $(s_n)$ of $(s_n^{'})$ such that for all $t, s\in \mathbb {R}$,

$$\begin{aligned} \lim _{n\rightarrow +\infty }K_{ij}(t-s+s_n)=K_{ij}^1(t-s), \quad \lim _{n\rightarrow +\infty }K_{ij}^1(t-s-s_n)=K_{ij}(t-s), \end{aligned}$$

and

$$\begin{aligned} \lim _{n\rightarrow +\infty }\psi (t+s_n)=\psi ^1(t), \quad \lim _{n\rightarrow +\infty }\psi ^1(t-s_n)=\psi (t). \end{aligned}$$

Define

$$\begin{aligned} \phi ^1:t\mapsto \int _{-\partial }^tK_{ij}(t-s)\psi ^1(s)ds. \end{aligned}$$

obviously,

$$\begin{aligned} \phi ^1(t+s_n)-\phi ^1(t)= & {} \int _{-\infty }^{t+s_n}K_{ij}(t-s+s_n)\psi (s)ds-\int _{-\infty }^{t}K_{ij}(t-s)\psi ^1(s)ds\\= & {} \int _{-\infty }^{t}K_{ij}(t-u)\psi (u+s_n)du-\int _{-\infty }^{t}K_{ij}(t-s)\psi ^1(s)ds\\= & {} \int _{-\infty }^{t}K_{ij}(t-u)|\psi (u+s_n)-\psi ^1(s)|ds\\= & {} \int _{-\infty }^{t} Me^{-(t-s)u}|\psi (u+s_n)-\psi ^1(s)|ds. \end{aligned}$$

In view of Lebesgue Dominated Convergence Theorem and (H2), we have for all $t\in \mathbb {R}$,

$$\begin{aligned} \lim _{n\rightarrow \infty }\phi (t+s_n)=\phi ^1(t). \end{aligned}$$

Similarly we have for all $t\in \mathbb {R}$,

$$\begin{aligned} \lim _{n\rightarrow \infty }\phi (t-s_n)=\phi (t), \end{aligned}$$

which implies that

$$\begin{aligned} \phi :t\mapsto \int _{-\infty }^t K_{ij}(t-s)g(x_{kl}(s))ds \end{aligned}$$

belongs to $AA(\mathbb {R},\mathbb {R})$. The proof of Lemma 3.1 is completed.

Define the nonlinear operator $\Phi$ by: for each $\varphi AA\in (\mathbb {R},\mathbb {R}^{m+n})$,

4 $$\begin{aligned} (\Phi \varphi )(t)= & \, col \Bigg \{\int _{-\infty }^te^{-\int _s^ta_{ij}(u)du}\Bigg [\sum _{C_{kl}\in N_r(i,j)} C_{ij}^{kl}(s)f(\varphi _{kl}(s-\tau _{kl}(s)))\varphi _{ij}(s)\nonumber \\&+\sum _{C_{kl}\in N_q(i,j)} B_{ij}^{kl}(s)\int _0^\infty K_{ij}(u)g(\varphi _{kl}(s-u))du \varphi _{ij}(s)+L_{ij}(s)\Bigg ]ds \Bigg \}. \end{aligned}$$

### **Lemma 3.2**

*If (H1–H3) are satisfied. Then* $\Phi$ *maps* $AA(\mathbb {R},\mathbb {R}^{m+n})$ *into itself.*

### *Proof*

First of all, let us check that $\Phi$ is well defined. By Lemma 2.1, we know that the space $AA(\mathbb {R},\mathbb {R}^{m+n})$ is translation invariant. Besides, by Lemmas 2.2 and Lemma 3.1, we can conclude that the function

5 $$\begin{aligned}&\Psi _{ij}: s\mapsto \sum _{C_{kl}\in N_r(i,j)} C_{ij}^{kl}(s)f(\varphi _{kl}(s-\tau _{kl}(s)))\varphi _{ij}(s)\nonumber \\&+\sum _{C_{kl}\in N_q(i,j)} B_{ij}^{kl}(s)\int _0^\infty K_{ij}(u)g(\varphi _{kl}(s-u))du \varphi _{ij}(s)+L_{ij}(s) \end{aligned}$$

belongs to $AA(\mathbb {R},\mathbb {R})$. Then (4) can be rewritten as

6 $$\begin{aligned} (\Phi \varphi )(t)=col \Bigg \{\int _{-\infty }^te^{-\int _s^ta_{ij}(u)du}\Psi _{ij} ds \Bigg \}. \end{aligned}$$

Let $(s_n^{'})$ be a sequence of real numbers. By (H4) we can extract a subsequence $(s_n)$ of $(s_n^{'})$ such that for all $t,s \in \mathbb {R}$,

7 $$\begin{aligned} \lim _{n\rightarrow +\infty }a_{ij}(t+s_n)=a_{ij}^1(t), \lim _{n\rightarrow +\infty }a_{ij}^1(t-s_n)=a_{ij}(t) \end{aligned}$$

and

8 $$\begin{aligned} \lim _{n\rightarrow +\infty }\Psi _{ij}(t+s_n)=\Psi _{ij}^1(t), \lim _{n\rightarrow +\infty }\Psi _{ij}^1(t-s_n)=\Psi _{ij}(t). \end{aligned}$$

Define

9 $$\begin{aligned} (\Phi ^1\varphi )(t):=\int _{-\infty }^te^{-\int _s^ta_{ij}^1(u)du}\Psi _{ij}(s)ds. \end{aligned}$$

Then

10 $$\begin{aligned}&(\Phi ^1\varphi )(t+s_n)-(\Phi ^1\varphi )(t)\nonumber \\&=\int _{-\infty }^{t+s_n}e^{-\int _s^{t+s_n}a_{ij}(u)du}\Psi _{ij}(s)ds -\int _{-\infty }^{t}e^{-\int _s^{t}a_{ij}^1(u)du}\Psi _{ij}^1(s)ds \nonumber \\&=\int _{-\infty }^{t+s_n}e^{-\int _{s-s_n}^{t}a_{ij}(u+s_n)du}\Psi _{ij}(s)ds -\int _{-\infty }^{t}e^{-\int _s^{t}a_{ij}^1(u)du}\Psi _{ij}^1(s)ds \nonumber \\&=\int _{-\infty }^{t}e^{-\int _{\theta }^{t}a_{ij}(u+s_n)du}\Psi _{ij}(\theta +s_n)d\theta -\int _{-\infty }^{t}e^{-\int _s^{t}a_{ij}^1(u)du}\Psi _{ij}^1(s)ds\nonumber \\&=\int _{-\infty }^{t}e^{-\int _{\theta }^{t}a_{ij}(u+s_n)du}\Psi _{ij}(\theta +s_n)d\theta -\int _{-\infty }^{t}e^{-\int _\theta ^{t}a_{ij}^1(u+s_n)du}\Psi _{ij}^1(\theta )d\theta \nonumber \\&\quad+\int _{-\infty }^{t}e^{-\int _{\theta }^{t}a_{ij}(u+s_n)du}\Psi _{ij}^1(\theta )d\theta -\int _{-\infty }^{t}e^{-\int _\theta ^{t}a_{ij}^1(u)du}\Psi _{ij}^1(\theta )d\theta \nonumber \\&=\int _{-\infty }^{t}e^{-\int _{\theta }^{t}a_{ij}(u+s_n)du}(\Psi _{ij}(s+s_n)-\Psi _{ij}^1(s))ds \nonumber \\& \quad -\int _{-\infty }^{t}\left( e^{-\int _\theta ^{t}a_{ij}(u+s_n)du}-e^{-\int _s^{t}a_{ij}^1(u)du}\right) \Psi _{ij}^1(s)ds. \end{aligned}$$

Applying the Lebesgue DominatedConvergence Theorem, we have

11 $$\begin{aligned} \lim _{n\rightarrow +\infty }(\Phi ^1(\varphi )(t+s_n))=(\Phi ^1\varphi )(t), \quad ~\text{ for } \text{ all } ~t\in \mathbb {R}. \end{aligned}$$

In a same way, we can prove that

12 $$\begin{aligned} \lim _{n\rightarrow +\infty }(\Phi ^1(\varphi )(t-s_n))=(\Phi \varphi )(t), ~\text{ for } \text{ all } ~t\in \mathbb {R}. \end{aligned}$$

Thus the function $(\Phi \varphi )$ belong to $AA(\mathbb {R},\mathbb {R})$. The proof of Lemma 3.2 is completed.

### **Theorem 3.1**

*If (H1–H5) are satisfied. Then system* (3) *has a unique almost automorphic solution in the region*

$$\begin{aligned} D=D(\varphi _0,\gamma )=\Bigg \{\varphi \in AA(\mathbb {R},\mathbb {R}^{m+n}), \, ||\varphi -\varphi _0||\le \frac{\gamma ||L||_\infty }{a^{-}(1-\gamma )}\Bigg \}, \end{aligned}$$

*where*

$$\begin{aligned} \varphi _0(t)=\left( \begin{array}{c} \int _{-\infty }^te^{-\int _s^ta_{11}(u)du}L_{11}(s)ds \\ \int _{-\infty }^te^{-\int _s^ta_{12}(u)du}L_{12}(s)ds \\ \vdots \\ \int _{-\infty }^te^{-\int _s^ta_{mn}(u)du}L_{mn}(s)ds \\ \end{array} \right) . \end{aligned}$$

### *Proof*

It is easy to see that

$$\begin{aligned} D=D(\varphi _0,\gamma )=\Bigg \{\varphi \in AA(\mathbb {R},\mathbb {R}^{m+n}), \quad ||\varphi -\varphi _0||\le \frac{\gamma ||L||_\infty }{a^{-}(1-\gamma )}\Bigg \} \end{aligned}$$

is a closed convex subset of $AA(\mathbb {R},\mathbb {R}^{m+n}).$ Then

13 $$\begin{aligned} ||\varphi _0(t)||= & {} \max _{ij\in \Lambda }\sup _{t\in \mathbb {R}}||\int _{-\infty }^te^{-\int _s^ta_{ij}(u)du}L_{ij}(s)ds||\nonumber \\= & {} ||L||_\infty \max _{ij\in \Lambda }\sup _{t\in \mathbb {R}}\int _{-\infty }^te^{-(t-s)a_{ij}^{-}}ds \nonumber \\= & {} \frac{||L||_\infty }{a^{-}}. \end{aligned}$$

Therefore, for any $\varphi \in D$ and by (13), we see easily that

14 $$\begin{aligned} ||\varphi ||\le ||\varphi -\varphi _0||+||\varphi _0||\le \frac{\gamma ||L||_\infty }{a^{-}(1-\gamma )}+ \frac{||L||_\infty }{a^{-}}=\frac{||L||_\infty }{a^{-}(1-\gamma )}. \end{aligned}$$

Now we prove that $\Phi$ is a self-mapping from *D* to *D*. In fact, for arbitrary $\varphi \in D$, it follows that

15 $$\begin{aligned}&||(\Phi \varphi )(t)-\varphi _0(t)||\nonumber \\&\quad =\max _{ij\in \Lambda }\sup _{t\in \mathbb {R}}\Bigg |\Bigg |\int _{-\infty }^te^{-\int _s^ta_{ij}(u)du}\Bigg \{\sum _{C_{kl}\in N_r(i,j)} C_{ij}^{kl}(s)f(\varphi _{kl}(s-\tau _{kl}(s)))\varphi _{ij}(s)\nonumber \\&\qquad +\sum _{C_{kl}\in N_q(i,j)} B_{ij}^{kl}(s)\int _0^\infty K_{ij}(u)g(\varphi _{kl}(s-u))du \varphi _{ij}(s)\Bigg \} ds \Bigg |\Bigg |\nonumber \\&\quad \le \max _{ij\in \Lambda }\sup _{t\in \mathbb {R}}\Bigg [\frac{\Big (\sum _{C_{kl}\in N_r(i,j)} |C_{ij}^{kl}(t)|L_f+\frac{M}{u}\sum _{C_{kl}\in N_q(i,j)}|B_{ij}^{kl}(t)|L_g\Big )\frac{||L||_\infty }{a^{-}(1-\gamma )} }{a^{-}}\Bigg ]||\varphi || \nonumber \\&\quad \le \max _{ij\in \Lambda }\sup _{t\in \mathbb {R}}\Bigg [\frac{\Big (\sum _{C_{kl}\in N_r(i,j)} |C_{ij}^{kl}(t)|L_f+\frac{M}{u}\sum _{C_{kl}\in N_q(i,j)}|B_{ij}^{kl}(t)|L_g\Big )}{a^{-}}\Bigg ]||\varphi || \nonumber \\&\quad \le \frac{\gamma ||L||_\infty }{a^{-}(1-\gamma )}, \end{aligned}$$

which implies that $(\Phi \varphi )\in D.$ Next, we prove the mapping $\Phi$ is a contraction mapping of *D*. In view of (H2), for any $\varphi ,\psi \in D,$ we have

16 $$\begin{aligned}&||(\Phi \varphi )(t)-(\Phi \psi )(t)|| \le \max _{ij\in \Lambda }\sup _{t\in \mathbb {R}}\int _{-\infty }^te^{-\int _s^ta_{ij}(u)du}\nonumber \\&\quad \times \,\Bigg \{\sum _{C_{kl}\in N_r(i,j)} |C_{ij}^{kl}(s)||f(\varphi _{kl}(s-\tau _{kl}(s)))\varphi _{ij}(s) -f(\psi _{kl}(s-\tau _{kl}(s)))\psi _{ij}(s)|\nonumber \\&\quad +\sum _{C_{kl}\in N_q(i,j)} |B_{ij}^{kl}(s)|\Bigg |\int _0^\infty K_{ij}(u)g(\varphi _{kl}(s-u))du \varphi _{ij}(s)\nonumber \\&\quad - \int _0^\infty K_{ij}(u)g(\psi _{kl}(s-u))du \psi _{ij}(s)\Bigg |\Bigg \} ds \nonumber \\&\le \max _{ij\in \Lambda }\sup _{t\in \mathbb {R}}\int _{-\infty }^te^{-\int _s^ta_{ij}(u)du}\nonumber \\&\quad \times \,\Bigg \{\sum _{C_{kl}\in N_r(i,j)} |C_{ij}^{kl}(s)|[M_f|\varphi _{ij}(s)-\psi _{ij}(s)|+L_f|\varphi _{kl}(s-\tau _{kl}(s))-\psi _{kl}(s-\tau _{kl}(s))|]\nonumber \\&\quad +\sum _{C_{kl}\in N_q(i,j)} |B_{ij}^{kl}(s)|\Bigg [\int _0^\infty |K_{ij}(u)|L_gdu |\varphi _{ij}(s)-\psi _{ij}(s)|\nonumber \\&\quad +\int _0^\infty |K_{ij}(u)|L_g|\varphi _{kl}(s-u)-\psi _{kl}(s-u)|\frac{||L||_\infty }{a^{-}(1-\gamma )} du \Bigg ]\Bigg \} ds \nonumber \\&\le \max _{ij\in \Lambda }\sup _{t\in \mathbb {R}}\int _{-\infty }^te^{-\int _s^ta_{ij}(u)du} \times \Bigg \{\sum _{C_{kl}\in N_r(i,j)} |C_{ij}^{kl}(s)|(M_f+L_f)\nonumber \\&\quad +\sum _{C_{kl}\in N_q(i,j)} |B_{ij}^{kl}(s)|\Bigg (1+\frac{||L||_\infty }{a^{-}(1-\gamma )}\Bigg )L_g \int _0^\infty |K_{ij}(u)|du\Bigg \} ds ||\varphi -\psi ||\nonumber \\&\le \max _{ij\in \Lambda }\sup _{s\in \mathbb {R}}\Bigg \{\frac{\Pi _{ij}}{a^{-}}\Bigg \}||\varphi -\psi ||, \end{aligned}$$

where

$$\begin{aligned} \Pi _{ij}=\sum _{C_{kl}\in N_r(i,j)} |C_{ij}^{kl}(s)|(M_f+L_f)+\sum _{C_{kl}\in N_q(i,j)} |B_{ij}^{kl}(s)|\Bigg (1+\frac{||L||_\infty }{a^{-}(1-\gamma )}\Bigg )L_g \int _0^\infty |K_{ij}(u)|du. \end{aligned}$$

Then it follows from (H4) that $\Phi$ is contracting operator in *D*. Thus there exists a unique almost automorphic solution $x^*\in D$ of (3) that is $\Phi (x^*)=x^*.$ The proof of Theorem 3.1 is completed.

## Exponential stability of almost automorphic solutions

In this section, we will obtain the exponential stability of the almost automorphic solutions of system (1).

### **Theorem 4.1**

*Suppose that (H1–H5) are fulfilled. If the condition (H6)*

$$\begin{aligned} a_{{ij}_s}^{-}- & {} \Bigg \{\Bigg [\sum _{C_{kl}\in N_r(i,j)} {C_{ij}^{kl}}^{+}(M_f+e^{\tau t}L_f)\\+&\sum _{C_{kl}\in N_q(i,j)} {B_{ij}^{kl}}^{+}\Bigg [L_g\int _0^\infty K_{ij}(u)du+ L_g\frac{||L||_\infty }{a^{-}(1-\gamma )}\int _0^\infty K_{ij}(u)e^{ u t}du\Bigg ]\Bigg \}>0 \end{aligned}$$

*holds, then the almost automorphic solution of system* (3) *in D* *is globally exponentially stable.*

### *Proof*

By Theorem 3.1, we know that (3) has an almost automorphic solution $x(t)=(x_{11}(t),x_{12}(t),\ldots ,$ $x_{mn}(t))^T$ with initial condition $\varphi (t)=(\varphi _{11}(t),\varphi _{12}(t),\ldots ,\varphi _{mn}(t))^T$. Suppose that $y(t)=(y_{11}(t),y_{12}(t),\ldots ,$ $y_{mn}(t))^T$ is an arbitrary solution of (3) with initial condition $\psi (t)=(\psi _{11}(t),\psi _{12}(t),\ldots ,\psi _{mn}(t))^T$. Denote $u(t)=(u_{11}(t),u_{12}(t),\ldots ,u_{mn}(t))^T$, where $u_{ij}(t)=y_{ij}(t)-x_{ij}(t), ij\in \Lambda .$ Set

17 $$\begin{aligned} \Upsilon _{ij}(t)= & \, t-a_{ij}+\sum _{C_{kl}\in N_r(i,j)} {C_{ij}^{kl}}^{+}(M_f+e^{\nu \tau }L_f)\nonumber \\+&\sum _{C_{kl}\in N_q(i,j)}{B_{ij}^{kl}}^{+}\Bigg [L_g\int _0^\infty K_{ij}(u)du+ L_g\frac{||L||_\infty }{a^{-}(1-\gamma )}\int _0^\infty K_{ij}(u)e^{\nu u}du\Bigg ]. \end{aligned}$$

Clearly, the functions $t\rightarrow \Upsilon _{ij}, ij\in \Lambda ,$ are continuous on $\mathbb {T}^{+}$ and by hypothesis (H6), $\Upsilon _{ij}(0)<0.$ Thus, there exists a sufficiently small constant $\nu$ such that $\Upsilon _{ij}(\nu )<0.$ Take an arbitrary $\varepsilon >0.$ Set

18 $$\begin{aligned} z_{ij}(t)=|x_{ij}^*(t)-x_{ij}(t)|e^{\nu t}. \end{aligned}$$

Then for all $ij\in \Lambda ,$ and for all $-\tau \le t\le 0,$ one has

19 $$\begin{aligned} z_{ij}(t)\le M<M+\varepsilon . \end{aligned}$$

Next, we shall prove that for all $t>0,$

20 $$\begin{aligned} z_{ij}(t)\le M+\varepsilon , \quad ij\in \Lambda . \end{aligned}$$

Suppose the contrary. Let us denote $A_{ij}=\{ t> 0, z_{ij} (t)> M + \varepsilon \}$. It follows that there exists $(ij)_0\in \Lambda$ such that $A_{(ij)_0}\ne \emptyset$. Let

21 $$\begin{aligned} t_{ij}=\left\{ \begin{array}{lc} \inf (A_{ij})~\{t>0, z_{ij}(t)>M+\varepsilon \}\ne \emptyset ,\\ +\infty ~~~~~~\{t>0, z_{ij}(t)>M+\varepsilon \}= \emptyset . \end{array}\right. \end{aligned}$$

Clearly $t_{ij} >0$ and for all $-\tau \le t\le t_{ij}$. Further, one has $z_{ij}(t)\le M+\varepsilon .$ Let us denote $t_{{ij}_s} = \min _{ij\in \Lambda }t_{ij}.$ It follows that $0<t_{{ij}_s}<+\infty .$ and for all $-\tau \le t\le t_{{ij}_s}.$ Note that

22 $$\begin{aligned} z_{{ij}_s}(t_{{ij}_s})= M+\varepsilon , D^{+}z_{{ij}_s}(t_{{ij}_s})\ge 0. \end{aligned}$$

Since $x_{ij}(.)$ and $x_{ij}^*(.)$ are solutions of (3), we get

23 $$\begin{aligned} 0\le & \, D^{+}z_{{ij}_s}(t_{{ij}_s})=D^{+}[|x_{ij}^*(t)-x_{ij}(t)|e^{\nu t}]_{t=t_{{ij}_s}}\nonumber \\= & \, e^{\nu t_{{ij}_s}} \left[ \nu |x_{ij}^*(t)-x_{ij}(t)|+\frac{D^{+}|x_{ij}^*(t)-x_{ij}(t)|}{dt}\right] \Bigg |_{t=t_{{ij}_s}}\nonumber \\= & \, |x_{{ij}_s}^*(t_{{ij}_s})-x_{{ij}_s}(t_{{ij}_s})|\nu e^{\nu t_{{ij}_s}}+e^{\nu t_{{ij}_s}} \text{ sgn } (x_{{ij}_s}^*(t_{{ij}_s})-x_{{ij}_s}(t_{{ij}_s}))\nonumber \\&\quad \times \Bigg \{-a_{{ij}_s}(t_{{ij}_s})(x_{{ij}_s}^*(t_{{ij}_s})-x_{{ij}_s}(t_{{ij}_s}))\nonumber \\&\quad +\sum _{C_{kl}\in N_r(i,j)} C_{{ij}_s}^{kl}(t_{{ij}_s})[f(x_{kl}^*(t_{{ij}_s}-\tau _{kl}(t_{{ij}_s})))x_{{ij}_s}^*(t_{{ij}_s})\nonumber \\&\quad -f(x_{kl}(t_{{ij}_s}-\tau _{kl}(t_{{ij}_s})))x_{{ij}_s}(t_{{ij}_s})]\nonumber \\&\quad +\sum _{C_{kl}\in N_q(i,j)} B_{{ij}_s}^{kl}(t_{{ij}_s})\Bigg [\int _0^\infty K_{{ij}_s}(u)g(x_{kl}^*(t_{{ij}_s}-u))du x_{{ij}_s}^*(t_{{ij}_s})\nonumber \\&\quad -\int _0^\infty K_{{ij}_s}(u)g(x_{kl}(t_{{ij}_s}-u))du x_{{ij}_s}(t_{{ij}_s})\Bigg ]\nonumber \\\le & \, |x_{{ij}_s}^*(t_{{ij}_s})-x_{{ij}_s}(t_{{ij}_s})|\nu e^{\nu t_{{ij}_s}}+e^{\nu t_{{ij}_s}}\Big [-a_{{ij}_s}(t_{{ij}_s})|x_{{ij}_s}^*(t_{{ij}_s})-x_{{ij}_s}(t_{{ij}_s})|\nonumber \\&\quad +\sum _{C_{kl}\in N_r(i,j)} |C_{ij}^{kl}(t_{{ij}_s})|[M_f|x_{ij}^*(t_{{ij}_s})-x_{ij}(t_{{ij}_s})|\nonumber \\&\quad +L_f|x_{kl}^*(t_{{ij}_s}-\tau _{kl}(t_{{ij}_s}))-x_{kl}(t_{{ij}_s}-\tau _{kl}(t_{{ij}_s}))|]\nonumber \\&\quad +\sum _{C_{kl}\in N_q(i,j)} |B_{ij}^{kl}(t_{{ij}_s})|\Bigg [\int _0^\infty |K_{ij}(u)|L_gdu |x_{ij}^*(t_{{ij}_s})-x_{ij}(t_{{ij}_s})|\nonumber \\&\quad +\int _0^\infty |K_{ij}(u)|L_g|x_{kl}^*(t_{{ij}_s}-u)-x_{kl}(t_{{ij}_s}-u)|\frac{||L||_\infty }{a^{-}(1-\gamma )} du\nonumber \\\le & \, (M+\varepsilon )(\nu -a_{{ij}_s}(t_{{ij}_s}))+\sum _{C_{kl}\in N_r(i,j)} |C_{ij}^{kl}(t_{{ij}_s})|\nonumber \\&\quad \times [M_f|z_{ij}(t_{{ij}_s})+e^{\nu \tau }L_fz_{kl}(t_{{ij}_s}-\tau _{kl}(t_{{ij}_s}))]\nonumber \\&\quad +\sum _{C_{kl}\in N_q(i,j)} |B_{ij}^{kl}(t_{{ij}_s})|\Bigg [\int _0^\infty |K_{ij}(u)|L_gdu z_{ij}(t_{{ij}_s})\nonumber \\&\quad +\int _0^\infty K_{ij}(u)e^{\nu u} L_g z_{kl}^*(t_{{ij}_s}-u)\frac{||L||_\infty }{a^{-}(1-\gamma )}du\nonumber \\\le & {} (M+\varepsilon )\Bigg [\nu -a_{{ij}_s}^{-}+\sum _{C_{kl}\in N_r(i,j)} {C_{ij}^{kl}}^{+}(M_f+e^{\nu \tau }L_f)\nonumber \\&\quad +\sum _{C_{kl}\in N_q(i,j)} {B_{ij}^{kl}}^{+}\Bigg [L_g\int _0^\infty K_{ij}(u)du+ L_g\frac{||L||_\infty }{a^{-}(1-\gamma )}\int _0^\infty K_{ij}(u)e^{\nu u}du\Bigg ]. \end{aligned}$$

It follows that

24 $$\begin{aligned}&\nu -a_{{ij}_s}^{-}+\sum _{C_{kl}\in N_r(i,j)} {C_{ij}^{kl}}^{+}(M_f+e^{\nu \tau }L_f)\nonumber \\&\quad +\sum _{C_{kl}\in N_q(i,j)} {B_{ij}^{kl}}^{+}\Bigg [L_g\int _0^\infty K_{ij}(u)du+ L_g\frac{||L||_\infty }{a^{-}(1-\gamma )}\int _0^\infty K_{ij}(u)e^{\nu u}du\ge 0. \end{aligned}$$

Then $\Upsilon _{ij}(\nu )\ge 0$ which contradicts the fact that $\Upsilon _{ij}(\nu )<0$. Thus we obtain that

25 $$\begin{aligned} z_{ij}(t)=|x_{ij}(t)-\varphi _{ij}(t)|\le (M+\varepsilon )e^{-\nu t}, \quad ~\text{ for } \text{ all }~ t>0. \end{aligned}$$

Note that $||x(t)-x_{ij}^*(t)||=\max _{ij\in \Lambda }|x_{ij}(t)x_{ij}^*(t)|,$ then letting $\varepsilon \rightarrow 0$, we obtain

26 $$\begin{aligned} |x(t)-x_{ij}^*(t)|\le M e^{-\nu t}, \quad ~\text{ for } \text{ all }~ t>0. \end{aligned}$$

which means that the almost automorphic solution of (3) is globally exponentially stable. The proof of Theorem 4.2 is completed.

### *Remark 4.1*

Shao (2008) studied the anti-periodic solutions of system (1) with the $B_{ij}(t)=0, a_{ij}(t)=a_{ij}$ and $\tau _{kl}=\tau (t)$. Peng and Huang (2009) investigated the existence and exponential stability of anti-periodic solutions for model (1) with $C_{ij}(t)=0$ and $a_{ij}(t)=a_{ij}.$ Zhao et al. (2010) considered anti-periodic solutions of model (1) with the $B_{ij}(t)=0$ and $\tau _{kl}=\tau (t)$. Peng and Wang (2011) analyzed the anti-periodic solutions for (1) with time-varying delays $\sigma _{ij}(t)$ in leakage terms. Zhou et al. (2006a) discussed the existence and stability of almost periodic solutions for model (1) with $C_{ij}(t)=0$. Li and Wang (2012) focused on the almost periodic solutions for model (1) with $C_{ij}(t)=0$ on time scales. In addition, there are many papers that have investigated almost periodic solutions or convergence behavior of the special form or a more general form of model (1). We refer the reader to (Zhao and Zhang 2008; Cai et al. 2008; Huang and Cao 2003; Ding et al. 2008; Liu and Huang 2006, 2007; Liu 2007, 2009a, b; Fan and Shao 2010; Liu et al. 2006; Shao et al. 2009; Xia et al. 2007; Zhou et al. 2006b; Liu and Ding 2014; Li and Wang 2012; Li et al. 2008; Meng and Li 2008; Li and Huang 2008). In this paper, we consider the almost automorphic solutions of (1), which complement with some previous studies in (Shao 2008; Peng and Huang 2009; Zhao et al. 2010; Peng and Wang 2013; Zhou et al. 2006a; Zhao and Zhang 2008; Cai et al. 2008; Huang and Cao 2003; Ding et al. 2008; Liu and Huang 2007; Liu 2007, 2009a, b; Fan and Shao 2010; Liu and Huang 2006; Liu et al. 2006; Shao et al. 2009; Xia et al. 2007; Zhou et al. 2006b; Liu and Ding 2014; Li and Wang 2012; Li et al. 2008; Meng and Li 2008; Li and Huang 2008).

### *Remark 4.2*

In Li and Yang (2014), authors considered the almost automorphic solutions for neutral type neural networks with delays in leakage on time ccales, in Abbas et al. (2014), authors considered the almost automorphic solutions for neural networks with impulses. All the methods can not be applied to this paper to obtained our results in this paper. Therefore our results are completely new.

## Numerical example

In this section, we will give an example to illustrate the feasibility and effectiveness of our main results obtained in previous sections. Considering the following shunting inhibitory cellular neural networks with time-varying delays

27 $$\begin{aligned} \left\{ \begin{array}{lc} \displaystyle {x}_{11}^{'}(t)=-a_{11}(t)x_{11}(t)+\sum _{C_{kl}\in N_r(1,1)} C_{11}^{kl}(t)f(x_{kl}(t-\tau _{kl}(t)))x_{11}(t)\\ \displaystyle \quad \quad +\sum _{C_{kl}\in N_q(1,1)} B_{11}^{kl}(t)\int _0^\infty K_{11}(u)g(x_{kl}(t-u))du x_{11}(t)+L_{11}(t),\\ \displaystyle {x}_{12}^{'}(t)=-a_{12}(t)x_{12}(t)+\sum _{C_{kl}\in N_r(1,2)} C_{12}^{kl}(t)f(x_{kl}(t-\tau _{kl}(t)))x_{12}(t)\\ \displaystyle \quad \quad+\sum _{C_{kl}\in N_q(1,2)} B_{12}^{kl}(t)\int _0^\infty K_{12}(u)g(x_{kl}(t-u))du x_{12}(t)+L_{12}(t),\\ \displaystyle {x}_{21}^{'}(t)=-a_{21}(t)x_{21}(t)+\sum _{C_{kl}\in N_r(2,1)} C_{21}^{kl}(t)f(x_{kl}(t-\tau _{kl}(t)))x_{21}(t)\\ \displaystyle \quad \quad +\sum _{C_{kl}\in N_q(2,1)} B_{21}^{kl}(t)\int _0^\infty K_{21}(u)g(x_{kl}(t-u))du x_{21}(t)+L_{21}(t),\\ \displaystyle {x}_{22}^{'}(t)=-a_{22}(t)x_{22}(t)+\sum _{C_{kl}\in N_r(2,2)} C_{22}^{kl}(t)f(x_{kl}(t-\tau _{kl}(t)))x_{22}(t)\\ \displaystyle \quad \quad \quad+\sum _{C_{kl}\in N_q(2,2)} B_{22}^{kl}(t)\int _0^\infty K_{22}(u)g(x_{kl}(t-u))du x_{22}(t)+L_{22}(t), \end{array}\right. \end{aligned}$$

where $$f(u)=0.5(|u+1|-|u-1|), K_{ij}=\cos \left( \frac{1}{2+\sin t+\sin \sqrt{2}t}\right)$$ and

$$\begin{aligned}&\left[ \begin{array}{cc} a_{11}(t) &{} a_{12}(t) \\ a_{21}(t) &{} a_{22}(t) \\ \end{array} \right] =\left[ \begin{array}{cc} 5+2\cos \sqrt{2}t &{} 7+2\cos \sqrt{3}t \\ 6+3\cos \sqrt{5}t &{} 4+2\cos \sqrt{3}t \\ \end{array} \right] ,\\&\left[ \begin{array}{cc} C_{11}(t) &{} C_{12}(t) \\ C_{21}(t) &{} C_{22}(t) \\ \end{array} \right] =\left[ \begin{array}{cc} 0.0002+0.0002\sin \sqrt{5}t &{} 0.0002+0.0001\sin \sqrt{3}t \\ 0.0002+0.0001\sin \sqrt{2}t &{} 0.0003+0.0001\sin \sqrt{3}t \\ \end{array} \right] ,\\&\left[ \begin{array}{cc} B_{11}(t) &{} B_{12}(t) \\ B_{21}(t) &{} B_{22}(t) \\ \end{array} \right] =\left[ \begin{array}{cc} 0.0003+0.0001\sin \sqrt{2}t &{} 0.0003+0.0001\sin \sqrt{3}t \\ 0.0002+0.0001\sin \sqrt{5}t &{} 0.0002+0.0001\sin \sqrt{5}t \\ \end{array} \right] ,\\&\left[ \begin{array}{cc} L_{11}(t) &{} L_{12}(t) \\ L_{21}(t) &{} L_{22}(t) \\ \end{array} \right] =\left[ \begin{array}{cc} 0.002+0.002\cos \sqrt{3}t &{} 0.003+0.002\cos \sqrt{7}t \\ 0.002+0.002\cos \sqrt{7}t &{} 0.001+0.002\cos \sqrt{3}t \\ \end{array} \right] . \end{aligned}$$

Let $r=q=1, \tau _{kl}(t)=0.005$. Then we get $L_f=L_g=M_g=M_f=1, a^{-}=2, ||L||_\infty =0.005, K_{ij}(t)\le e^{-t}, M=u=1, \tau =0.005$ and

$$\begin{aligned}&\left[ \begin{array}{cc} \sum _{C_{kl}\in N_1(1,1)} {C_{11}^{kl}}^{+} &{} \sum _{C_{kl}\in N_1(1,2)} {C_{12}^{kl}}^{+} \\ \sum _{C_{kl}\in N_1(2,1)} {C_{21}^{kl}}^{+} &{} \sum _{C_{kl}\in N_1(2,2)} {C_{22}^{kl}}^{+} \\ \end{array} \right] =\left[ \begin{array}{cc} 0.0014 &{} 0.0014\\ 0.0014 &{} 0.0014 \\ \end{array} \right] ,\\&\left[ \begin{array}{cc} \sum _{C_{kl}\in N_1(1,1)} {B_{11}^{kl}}^{+} &{} \sum _{C_{kl}\in N_1(1,2)} {B_{12}^{kl}}^{+} \\ \sum _{C_{kl}\in N_1(2,1)} {B_{21}^{kl}}^{+} &{} \sum _{C_{kl}\in N_1(2,2)} {B_{22}^{kl}}^{+} \\ \end{array} \right] =\left[ \begin{array}{cc} 0.0016 &{} 0.0016 \\ 0.0016 &{} 0.0016 \\ \end{array} \right] . \end{aligned}$$

Hence

$$\begin{aligned}\gamma &=\max _{ij\in \Lambda }\sup _{t\in \mathbb {R}}\Bigg \{\frac{\sum _{C_{kl}\in N_1(i,j)} |C_{ij}^{kl}(t)|L_f+\frac{M}{u}\sum _{C_{kl}\in N_1(i,j)}|B_{ij}^{kl}(t)|L_g}{a^{-}}\Bigg \}\\ &\le \frac{0.0014+0.0016}{2}=0.0015 <1,\\ \frac{||L||_\infty }{a^{-}(1-\gamma )}&=\frac{0.005}{1(1-0.0015)}=\frac{10}{17}<1,\\ \Pi _{ij}&=\sum _{C_{kl}\in N_1(i,j)} |C_{ij}^{kl}(s)|(M_f+L_f)\\&\quad +\sum _{C_{kl}\in N_1(i,j)} |B_{ij}^{kl}(s)|\Bigg (1+\frac{||L||_\infty }{a^{-}(1-\gamma )}\Bigg )L_g \int _0^\infty |K_{ij}(u)|du \\ &\le 0.0014\times 2+0.0016\times 0.6=0.00376,\\ \max _{ij\in \Lambda }\sup _{s\in \mathbb {R}}\left\{\frac{\Pi _{ij}}{a^{-}}\right\}&=0.00188<1, \end{aligned}$$

$$\begin{aligned}& a_{{ij}_s}^{-}-\Bigg [\sum _{C_{kl}\in N_1(i,j)} {C_{ij}^{kl}}^{+}(M_f+e^{\tau t}L_f)-\sum _{C_{kl}\in N_1(i,j)} {B_{ij}^{kl}}^{+}\Bigg [L_g\int _0^\infty K_{ij}(u)du\nonumber \\&\quad\quad + L_g\frac{||L||_\infty }{a^{-}(1-\gamma )}\int _0^\infty K_{ij}(u)e^{ ut}du\Bigg ]=1.000624>0. \end{aligned}$$

**Fig. 1:**
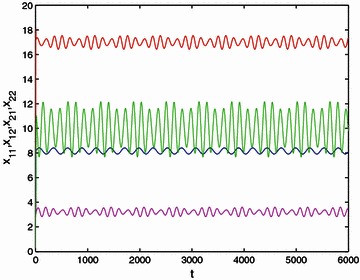
Time response of state variables $x_{ij}(i,j=1,2),$ where the* red line* stands for $x_{11},$ the* magenta line *stands for $x_{12},$, the* blue line* stands for $x_{21}$ and the* green line* stands for $x_{22}$

Thus all assumptions in Theorems 4.1 and 4.2 are fulfilled. Thus we can conclude that (27) has an almost automorphic solution, which is globally exponentially stable. The results are verified by the numerical simulations in Fig. 1.

## Conclusions

In this paper, we consider a class of shunting inhibitory cellular neural networks with time-varying delays. Some sufficient conditions for the existence and exponential stability of almost automorphic solutions for the shunting inhibitory cellular neural networks with time-varying delays have been established. It is shown that the time delay has no effect on the existence of almost automorphic solutions for system (1) but has important effect on the global exponential stability of almost automorphic solutions for system (1). To the best of our knowledge, it is the first time to deal with the almost automorphic solution for the shunting inhibitory cellular neural networks with time-varying delays. Moreover, our criteria are easy to check and apply in practice and are of prime importance and great interest in many application fields and the designs of networks. Our results complement with some previous ones. The method of this paper can be applied directly to many other neural networks, such as BAM neural networks, Hopfield neural networks and so on.
